# Reconstructing and counting genomic fragments through tagmentation-based haploid phasing

**DOI:** 10.1038/s41598-021-97852-w

**Published:** 2021-09-23

**Authors:** Patrick P. T. Leong, Aleksandar Mihajlović, Nadežda Bogdanović, Luka Breberina, Larry Xi

**Affiliations:** 1 Digenomix Corp, Hayward, CA USA; 2 Department of Informatics, Technische Universität München, Munich, Germany; 3 Faculty of Mathematics, University of Belgrade, Belgrade, Serbia; 4 Faculty of Chemistry, University of Belgrade, Belgrade, Serbia

**Keywords:** Biochemistry, Biological techniques, Biotechnology, Computational biology and bioinformatics, Molecular biology

## Abstract

Single-cell sequencing provides a new level of granularity in studying the heterogeneous nature of cancer cells. For some cancers, this heterogeneity is the result of copy number changes of genes within the cellular genomes. The ability to accurately determine such copy number changes is critical in tracing and understanding tumorigenesis. Current single-cell genome sequencing methodologies infer copy numbers based on statistical approaches followed by rounding decimal numbers to integer values. Such methodologies are sample dependent, have varying calling sensitivities which heavily depend on the sample’s ploidy and are sensitive to noise in sequencing data. In this paper we have demonstrated the concept of integer-counting by using a novel bioinformatic algorithm built on our library construction chemistry in order to detect the discrete nature of the genome.

## Introduction

In recent years, attention has been extended from exonic single nucleotide variations to underlying copy number variations of genes^1^. It has been postulated that gene copy number variation is a result of a cellular response intended to increase survivability under stress inducing conditions. Such copy number changes can either promote survival of the cell under adverse conditions, or can induce abnormal behavior resulting in carcinogenesis, metastasis, or drug resistance^2–6^. The dynamics and heterogeneity of genomic copy number changes in response to adverse stresses has been the focus of many investigations to elucidate the forces that shape genomes in eukaryotic cells and likely influence karyotypic evolution in cancer cells^4–7^.

Traditionally, molecular cytogenetic techniques such as fluorescence in situ hybridization (FISH) and spectral karyotyping (SKY) are the chosen methods to determine discrete gene copy numbers^8,9^. However, these techniques suffer from low throughput, low resolution, high labor cost, and often higher error rates^10^. More recently, several NGS and NGS-based single-cell DNA sequencing technologies with improved throughput have been reported^11^. The copy number of the genes are calculated in secondary analyses by binning the number of mapped reads across the genome, grouping the bins into segments of similar quantities, then using these non-integer values and statistical methods to infer a discrete copy number for each segment. The performance of these algorithms are reported to be impacted to their tuning parameters, sequencing noise, and the ploidy of genomes. As a result, the precision can range from 0 to 90% in the worst cases using simulated data^12^. Previously, we published a novel single-cell DNA sequencing scheme^13,14^, dubbed Barcodes_in_Genome_sequencing (BIGseq), which has the potential to detect the integer copy numbers at single base resolution. In order to reach that potential, the coverage of every haploid molecule in the single cell library needs to be high enough and a secondary analysis algorithm can process the library data properly. In this work, we have improved the median coverage to 79% for every haploid DNA molecule at less than 8 × sequencing depth. We have also developed a novel secondary analysis algorithm to reconstruct every haploid DNA molecule by piecing together fragments based on the unique deterministic nature of library construction. In this paper, we demonstrate that the number of DNA molecules in a vessel can be reliably counted in integers, demonstrating the feasibility to count discrete gene copy numbers within single cells.

## Methods

### Construction of Tn5 transpososome

In order to overcome the limitation that Nextera transpososome can only recover 50% of tagmented fragments at maximum^15,16^, we constructed a Tn5 transpososome with identical transposomes ends by loading transposase (Cat. No. EMQZ1422) from Creative Biogene, Shirley, NY, with a single duplex formed by NEX8a (5′-CAGAGATGTGTATAAGAGACAG-3′) and Tn5Up (5′-Phos-CTGTCTCTTATACACATCT-3′) according to the protocol provided by the manufacturer. This transpososome was let to sit at 4 °C overnight before it was used for library construction.

### Construction of primary library, library amplification, and sequencing

The constructed transpososome was used to tagment about 3 femtograms of yeast genomic DNA (ATCC Cat. No. 9763), which was serially diluted from a stock of 6 ng/μL, to generate a primary library^13^. The transposase was removed from DNA by Protease K (New England Biolabs, Cat. No. P8107S) treatment and then Protease K was denatured by heat. The primary library was sequentially amplified by Phusion Hot Start II High-Fidelity PCR Master Mix (Thermo Fisher Scientific Cat. No. F565L) with NEX8a for three cycles, and then together with Illumina Read1 (5′-TCGTCGGCAGCGTCAGATGTGTATAAGAGACAG-3′) and Read2 (5′GTCTCGTGGGCTCGGAGATGTGTATAAGAGACAG-3′) primer pair for 20 cycles, both with 30-min extension time at 65 °C. The amplified library was cleaned with AMPure (Beckman Coulter Cat. No. A63882) beads twice and eluted in 20 μL 10 Tris buffer, 8 μL of the eluents was taken to be barcoded in a 20 μL reaction in 6 cycles. The barcoded libraries were cleaned with AMPure beads twice and loaded onto MiSeq (Illumina, Cat. No. MS-102-3001) for sequencing 80 bases for Read1 and 70 bases for Read2, after quantitation following the manufacturer’s protocol^14^.

### Adapter trimming

The MiSeq output files were first passed through the Illumina primary analysis pipeline to remove the majority of the primer and adapter sequences. The resulting paired-end FASTQ files were evaluated for the percentage of reads from yeast genome relative to other known genomes using FastQ Screen (https://www.bioinformatics.babraham.ac.uk/projects/fastq_screen/). This served as a check for yield and contamination. The same FASTQ files were sent through Trim Galore (http://www.bioinformatics.babraham.ac.uk/projects/trim_galore/), which used Cutadapt (https://cutadapt.readthedocs.io/en/stable/index.html)^17^ and FastQC (https://www.bioinformatics.babraham.ac.uk/projects/fastqc/) as software engines, for removal of any remaining Illumina Read 1 and Read 2 primer adapters. The Trim Galore stringency for adapter matching was set at 15 bp (default 1); minimum Phred score was set at 20 (default); and any read shorter than 25 bp after quality or adapter trimming was discarded. All other Trim Galore parameters were at their default values.

### Mapping

The Trim Galore processed read sequences in FASTQ format were mapped to the reference yeast genome (GenBank assembly accession: GCA_000146045.2) using the BOWTIE2 aligner^18^ with default parameters except the maximum fragment length for valid paired-end alignment (-X option) was set at 1000. The aligner generated a raw BAM file. Our in-house Python scripts would reconstructed fragments in BAM format to represent an amplified library (Column 2 of Table 1, the number of reads is shown in Column 4). During this process, two reads would merge if the fragment length was shorter than or equal to 150 bases, while a gap between the two reads was filled in with the reference genome sequences when the fragment length was greater than the combined read lengths of 150 bases. The file was then deduplicated to remove PCR duplications to represent the primary library (Column 3 of Table 1, the number of reads is shown in Column 5).

**Table 1:** Sample names, file names, and sequencing statistics of BIGseq on fractions of yeast genome DNA.

Sample name	Raw BAM file	Library-representing BAM file	Total fragments mappable to yeast before dedupe	Fragment number with unique UFI after dedupe	Average duplication rate of fragments	Average length of fragments	Percentage genome covered by fragments
6Y5j	6Y5j_S37_Ckite.bam	6Y5j_S37_Ckite_pemerged_deduped.bam	79,567	12,436	6.4	233.8	8.8
6Y5l	6Y5l_S38_Ckite.bam	6Y5l_S38_Ckite_pemerged_deduped.bam	103,722	13,930	7.4	233.1	9.2
6Y5n	6Y5n_S39_Ckite.bam	6Y5n_S39_Ckite_pemerged_deduped.bam	57,997	12,488	4.6	237.3	8.6
6Y5p	6Y5p_S40_Ckite.bam	6Y5p_S40_Ckite_pemerged_deduped.bam	88,350	13,618	6.5	225.0	8.7
6Y6p	6Y6b_S41_Ckite.bam	6Y6b_S41_Ckite_pemerged_deduped.bam	28,875	9232	3.1	225.4	6.6
6Y6d	6Y6d_S42_Ckite.bam	6Y6d_S42_Ckite_pemerged_deduped.bam	40,055	9984	4.0	220.8	7.1
6Y6f	6Y6f_S43_Ckite.bam	6Y6f_S43_Ckite_pemerged_deduped.bam	50,127	9760	5.1	220.7	7.0
6Y6h	6Y6h_S44_Ckite.bam	6Y6h_S44_Ckite_pemerged_deduped.bam	48,896	9440	5.2	222.3	6.6

### Reconstruction DNA molecules

We modified a web browser-based Integrative Genomics Viewer (IGV)^19,20^ to display how various mapped fragments created during the tagmentation process can be re-assembled into their originating DNA molecules by concatenating them at their 9-base overlapping ends, or “junctions” as we refer to them. Specifically, the BIGseq algorithm used an exclusivity and greediness approach to re-assemble unique DNA molecules over a given region of the genome: the algorithm started from a fragment mapped to the very 5′ end of the reference genome and scanned towards the 3′ direction looking for an adjacent fragment that shares a 9-base junction with the first fragment. Once the second fragment was identified, the algorithm semantically joins two fragments to form a contiguous structure referred to as an “island”. The greediness approach would attempt to extend the length of the island as long as there are adjacent fragments that share 9 base junctions with it. In case a third mapped fragment that overlapped with the first island but cannot be joined with the island through 9-base junction, the exclusivity rule would assign the third fragment to a new molecule. Then, the algorithm would continue to the next fragment downstream and apply greediness rule to the existing DNA molecules by packing fragments and islands wherever they were logically possible (more detailed examples in “Results” section).

### In-silico simulation

Additionally, to provide a calibration background on whether certain regions of the genome were more prone to alternative alignments by the sequence aligner, from the yeast reference genome, we simulated a haploid with fixed-size read-pairs of read length 50 bp and insert sizes varied over the range 35–150 bp. Each read had a 9-base overlap with its adjacent neighbors. We then used the same BOWTIE2 aligner to map back these simulated reads. The read length and insert size range was intentionally kept small to test the ability of the aligner to correctly map the reads. If there was no simulated read being mis-mapped over a specific region of the genome, this gave us confidence that the real sample reads being mapped there were correctly mapped.

### Base preferences at junctions

Overlapping sequences with 8-, 9-, and 10-bases and their surrounding sequences were extracted and analyzed at the sequence logo site for the relative frequencies of every base at every position (https://weblogo.berkeley.edu/logo.cgi).

## Results

### High coverage and specific mapping lead to the identification of archipelagos reflecting input DNA

Integer counting of haploids in BIGseq is based on the deterministic property of tagmentation: a long stretch of double-stranded DNA can generate only one unique pattern upon tagmentation. The pattern can be determined bioinformatically as long as each fragment is mapped accurately and the junctions, 9-base overlaps between fragments created by transpososomes^21,22^, is identified correctly.

When mapping in-silico generated reads, we discovered 3% of these reads were mismapped by the BOWTIE2 aligner. Such mismapped reads typically landed to some specific locations of the genome. For comparison, a significantly higher percentage of reads were found to be mismapped by BWA-MEM aligner^23^. Thus, BOWTIE 2 was selected for our work.

Table 1 summarizes the names, corresponding files, and key parameters of the actual yeast samples. The average lengths of fragments are slightly over 220 bases. The duplication rate, which is defined as the ratio between the total fragment number and the fragment number with unique fragment index (UFI)^13^, is between 3.1 and 7.4. The percentage of the genome covered by the fragments of each library ranges from 6.6 to 9.2%.

**Figure 1:**
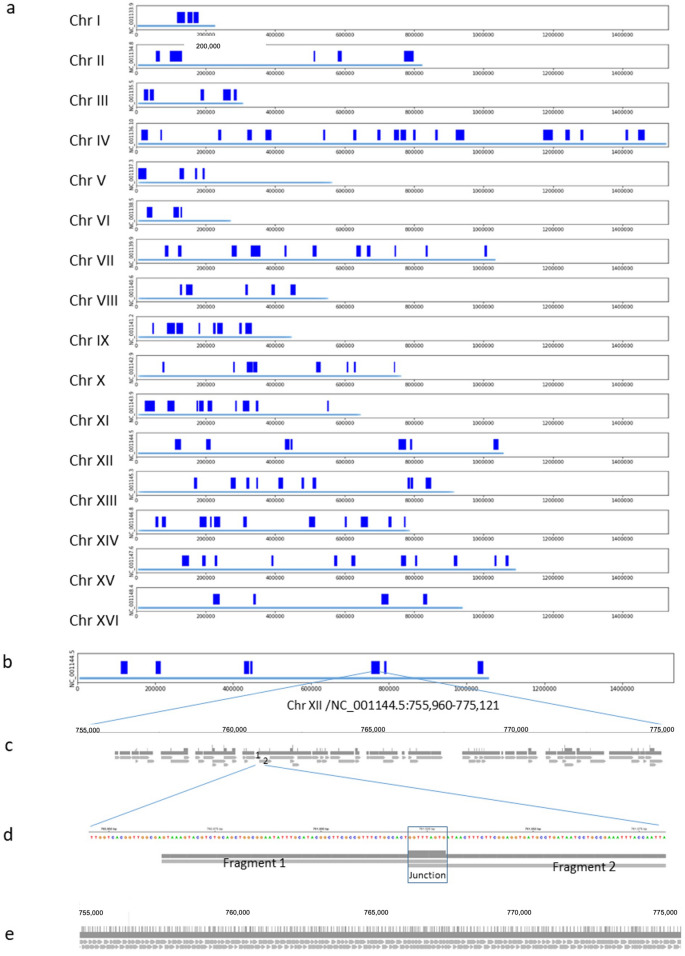
Archipelagos identified in sample 6Y5j. (**a**) Distribution of identified archipelagos (dark blue bar) is displayed for 16 chromosomes of 6Y5j. The size of each chromosome is labelled with a light blue line above the coordinate. (**b**) Archipelagos of Chr XII/NC_001144.5 are presented. (**c**) An archipelago in the region of Chr XII/NC_001144.5 755,960–775,121 is zoomed in to show fragments after deduplication. (**d**) The 9-base junction and its surrounding regions between Fragments 2 and 3 in (**c**) are further zoomed in. (**e**) Simulation of tagmentation of the same genome region as in (**c**) are presented to show the perfect tiling. The graphs were generated with Microsoft PowerPoint and IGV (https://software.broadinstitute.org/software/igv/).

The sequencing files were visualized on the modified web browser-based IGV^19,20^. We observed that most of the reads were in clusters, which we call archipelagos, while nearly 90% of genome regions were almost blank (Fig. 1a), with scattered lonely fragments that consisted of 10% of total deduplicated fragments and covered less than 1% of the blank regions of the entire genome. We manually inspected every one of the solo fragments. Most, if not all, were smaller than two hundred bases, and do not have a possible second mapping site. We hypothesize that they were from low-level cross-contamination. Because the frequency of these fragments was very low and their appearance in all the samples was randomly distributed, their existence did not significantly affect our data processing on archipelagos.

Figure 1a is a screenshot of a representative archipelagos, presented in blue bars, of sample 6Y5j of all chromosomes excluding mitochondrial DNA. 9.3% of genome was covered by 129 archipelagos, ranging from 1 to 30 Kb, with a median size of 7.9 Kb and a median coverage of 79%. We found that the average GC% of all archipelagos was indistinguishable from that of blank regions outside the archipelagos, and the average GC% of the library-covered regions was indistinguishable from that of uncovered regions within archipelagos, suggesting low GC bias in the library construction process. The high coverage rates within archipelagos foretell that there is a low chance that we might missed any archipelagos. Figure 1b–e depict a representative result of a library-representing file. The whole Chr XII/NC_001144.5 in BED format is displayed in Panel B, with nine archipelagos shown in blue bars. Among these nine archipelagos, a 19-Kb archipelago located in the region Chr XII/NC_001144.5) of 755,960–775,121 is zoomed in Panel C using a BAM file. This archipelago was made of a total of 75 fragments, with 36 junctions. One of the junctions is zoomed in Panel D. An in-silico mapping simulation of random tagmentation of a haploid covering the same region as Panel C is exhibited in Panel E. A perfect tiling pattern of this simulation is conspicuous, and it serves as an indicator of high specificity in mapping.

### Improved junction identification by trimming remaining primer sequences

Trim Galore (https://github.com/FelixKrueger/TrimGalore) identified about 0.1% of the FASTQ reads output from the Illumina primary processing pipeline still having remnant primer sequences and hence trimmed them. It also recognized that no more than 0.5% of the reads had Phred score less than 20 and they were discarded. Since we set the Trim Galore’s stringency for adapter matching to a minimum of 15 bases to avoid false positives, it is possible that some small pieces of the primer sequence still remain attached to the reads. Indeed we identified 12 cases of missed junctions resulting from incomplete removal of primer sequences. A case in point is the 12-base overlap presented at Chr XIV/NC_001146.8: 658,350–658,629. Of these 12 bases, only 9 bases truly overlap, while the three bases at the very 5′ end of the downstream fragment, CAG, are not shared with the upstream fragment, nor are they present in the reference genome (Fig. S1). This subsequence was identified to be the last three bases of primers used in PCR and it was removed to restore the real junction.

### The GC preference of Tn5 and the identification of uncanonical junctions

**Figure 2:**
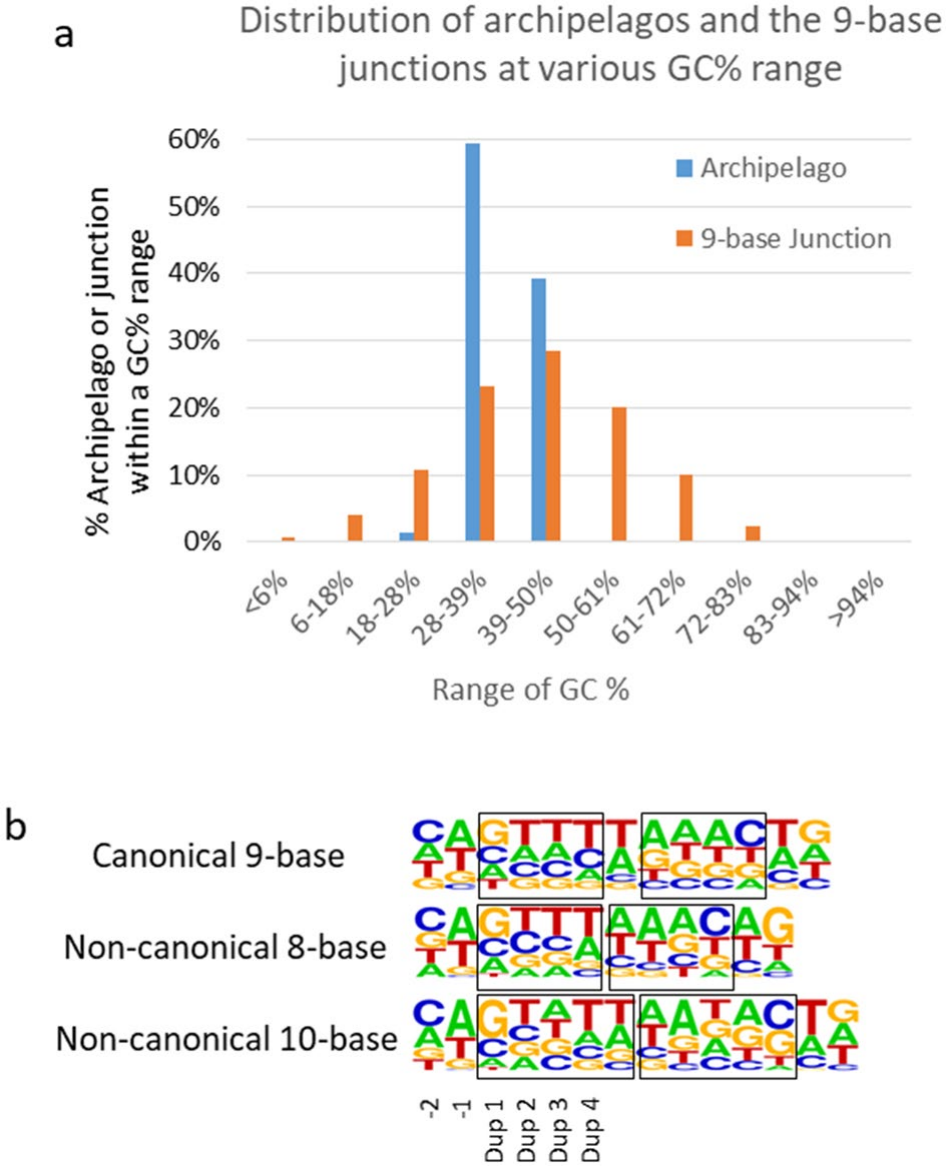
GC% of Archipelagos and the junction regions and base preference in the region at the transpososome cutting site. (**a**) GC distribution of Archipelago (blue) and GC distribution of the 9-base junction, at various range of GC%. (**b**) Base preference at 6 positions of top strand surrounding the cutting site for 9-base canonical and 8- and 10-base uncanonical cuts presented by sequence logo. The symmetry of the cutting site is marked by the two boxes. The six bases on the top strand flanking the cutting site in the direction from outside to the inside of the left monomer of the transposase are numbered as: -2,-1, Dup1, Dup2, Dup3 and Dup4, where the cut was made between -1 and Dup1. The graphs were generated by Microsoft Excel, Microsoft PowerPoint and weblogo (https://weblogo.berkeley.edu/logo.cgi).

The transpososome was reported to show biases in choosing its cutting sites^24,25^. If the bias is strong, it could be detrimental to the high coverage requirement of BIGseq. Incongruously, no GC bias was observed in the Nextera libraries^15^. Consistent with both sides of the earlier findings, our libraries had similar GC profiles to that of the whole yeast reference genome. At the same time, the junctions showed a higher GC average than the average of the genome while exhibiting extreme broad GC distribution (Fig. 2a). The indiscriminating cutting by Tn5 ensured the high coverage of our libraries.

Tn5 transposase is a homodimer, exhibiting twofold axis of symmetry with each monomer containing one active center^22^. We found that our transpososome preferred G immediately inside the cutting site (the Dup1 position), and A/T immediately outside the cutting site (− 1); it slightly preferred at − 2 position, while it is unscrupulous at the rest of the positions (Fig. 2b).

We discovered that in addition to 1889 9-base junctions in Sample 6Y5j, there were 97 8-base, and 139 10-base overlaps, respectively. The occurrence of 8- or 10-base overlaps was higher than the random chance. Ten-base junction was reported more than 30 years ago^26^ and has been rarely cited since. The base preferences exhibited by both 10- and 8-base junctions around the cutting site which was similar to those of the 9-base junctions (Fig. 2b) lead us to accept that Tn5 had at least two uncanonical cuts. Uncanonical cuts shed light on the reaction mechanisms of transpososome and the inclusion of uncanonical junctions helps credible reconstruction of the input molecules by BIGseq as shown in the following sections.

### Reconstruction of mono-ploidic molecule and identifications of artifacts caused by defective Tn5

**Figure 3:**
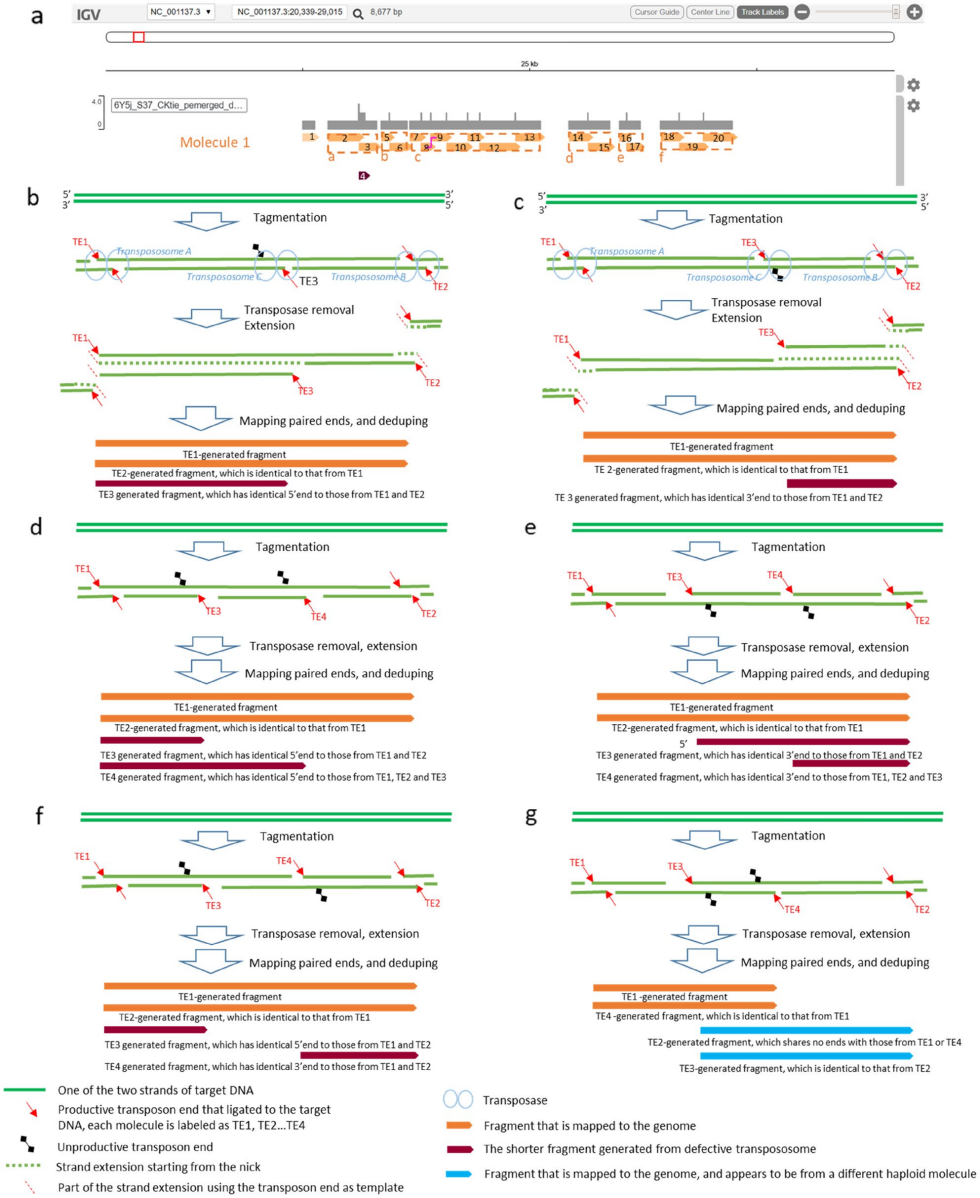
Defective transpososomes leads to artifacts. (**a**) Nineteen fragments in the archipelago of Chr V/NC_001137.3 22,504 to 27,231 are reconstructed into one molecule. The seemingly lone Fragment 4 led to the discovery of defective transpososomes. (**b–g**) are Defective Scenarios 0/1, 1/0, and Complex Scenarios 00/11, 11/00, 01/1/0, and 10/01 respectively. The graphs were generated with Microsoft PowerPoint and IGV (https://software.broadinstitute.org/software/igv/).

The first step of the BIGseq pipeline to reconstruct DNA molecules is to chain neighboring fragments through junctions to form larger contigs, referred to as islands. Then islands and fragments are assigned to each molecule, a process referred as phasing, by following the rules of exclusivity and greediness. Exclusivity requires that any fragment is allowed to belong to only one molecule, and two overlapping fragments must belong to separate molecules unless they share a junction of 8-, 9-, or 10-base. Greediness asks to assign as many islands and fragments as possible to the first molecule, then to the second, then the third, etc., until all islands are exhausted. As each library is derived from a pool of DNA fragments of about 10 kb long and of total quantity of 10% of the genome, the majority of the archipelagos must have been mono-ploid, i.e., most of the archipelagos were made of only one molecule, a case in the point was the 4.8 Kb archipelago located at Chr V/NC_001137.3: 22,504-27,231 (Fig. 3a), this molecule was 82% covered by 20 fragments. The reconstruction process started with chaining 18 fragments into six non-overlapping islands (a–f), then assigning them to Molecule 1. The fragments in islands were depicted in a darker color and arranged alternatively between two lanes in the modified IGV. Lastly, the unchained Fragment 1, shown in a lighter tone, joined islands a–f to complete the reconstruction of Molecule 1. By the rule of exclusivity, Fragment 4 was temporarily set to be the lone candidate for the second molecule.

We noticed that Fragment 4 had a 5′ end identical to Fragment 3, and both shared a 9-base junction with Fragment 2. Analyzing archipelagos of Sample 6Y5j revealed that quite a few fragments shared identical 5′ or 3′ ends. After examining the raw BAM files, we ruled out that Fragment 4 was a product of sequencing or alignment errors. Because the sequence within Fragment 3 shared no significant similarity to the sequences of the primers used in PCR or sequencing, it is unlikely that Fragment 4 was a PCR by-product resulting from mispriming on Fragment 3. Based on the sharing junction, we proposed a novel hypothesis that Fragment 4 resulted from an unreported defective Tn5 transpososome, of which one of the two reaction centers failed to make a nick to complete tagmentation. As Fragment 4 was identified as an artifact, this archipelago was reconstructed to be one molecule.

A detailed mechanism is proposed in Fig. 3b to account for our observation: Transpososome made a single tagmentation at the bottom strand, a scenario denoted as “0/1”, where “0” represents the defective center and “1” represents the productive center of dimeric Tn5 transposase. The digits above and below “/” represent the top and the bottom strands, respectively. During the extension step, the strands that ligated to transposon end molecules—TE1, TE2, and TE3—will extend from 3′ end to become functional templates for the next round, eventually generating two indistinguishable long pieces and a short piece sharing the 5′ end. In a similar fashion, a 1/0 scenario will generate a short fragment sharing the 3′ ends with two indistinguishable longer fragments (Fig. 3c).

If our hypothesis holds true, we can expect that there will be four more complex patterns emerging when two defective transposomes react side by side, as described below:

#### Complex scenarios 00/11 and 11/00

Where two defective reaction centers are adjacent to one another on the top strand (Fig. 3d) or on the bottom 5 strand (Fig. 3e) respectively, resulting in four fragments (three unique sequences of varying lengths) sharing identical 5′ end or 3′ ends respectively.

#### Complex scenario 01/10

Where the first defective reaction center is upstream on the top strand and the second defective reaction center is downstream on the bottom strand, resulting in four fragments, two shorter fragments with one sharing 5′ end and the other sharing 3′ end with the other two identical longer fragments (Fig. 3f).

#### Complex scenario 10/01

Where the first defective reaction center is upstream on the bottom strand, and the second defective reaction center is downstream on the top strand, leading to two overlapping fragments (Fig. 3g).

Just as we predicted, we identified a significant amount of instances of these complex artifacts. Some of the examples are shown in Fig. S2. These complex scenarios further reinforced our hypothesis. As we implemented an algorithm to eliminate these artifacts, the identified archipelago calls appeared to be cleaner and cohesive, as shown in the next section.

We should point out that Complex Scenario 10/01 generates two overlapping fragments, a pattern that is similar to the result if they came from two separate molecules (Fig. S2D). We are still in the process of developing algorithms to call this complex scenario. Meanwhile, we are engineering Tn5 to minimize the chance of defective reactions.

It is worth pointing out that Fragment 8 and Fragment 9 (Fig. 3a) share an 8-base junction, a point we discussed in previous section. If this junction was not recognized, then Fragments 9, 10, 11, 12, and 13 would have had to be in Molecule 2, leaving a big gap in Molecule 1, an unlikely scenario under our experimental condition.

### Reconstruction and counting of two DNA molecules by BIGseq

**Figure 4:**
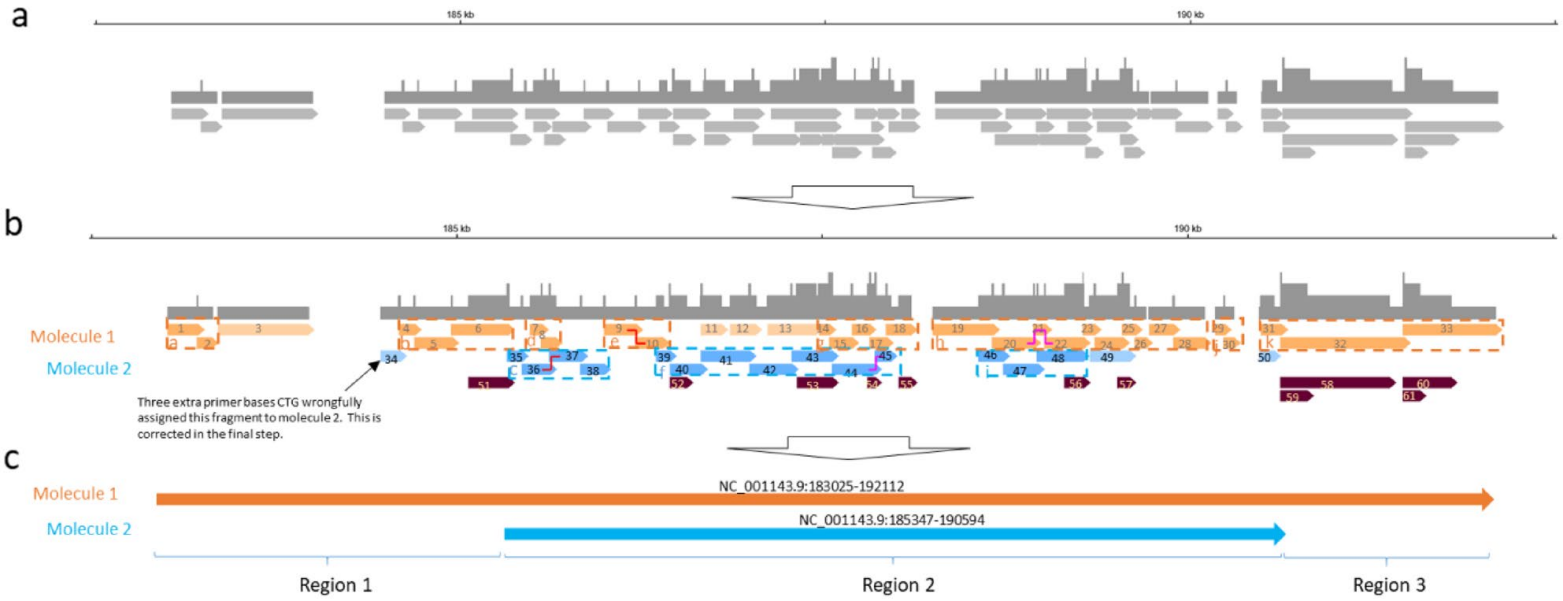
The BIGseq algorithm to identify two molecules. (**a**) Boundary of an archipelago is set. (**b**) Two molecules and redundant fragments are called. (**c**) Final presentation of two molecules. The graphs were generated with Microsoft PowerPoint and IGV (https://software.broadinstitute.org/software/igv/).

With the identification of the artifacts mentioned in the previous sections, we are able to reconstruct molecules in more complex situations as shown in Fig. 4. Panel A shows an archipelago identified in the region of Chr XI/NC_001143.9:183025–192112, with 61 deduplicated fragments. Among these 61 fragments, eleven fragments (Fragment 51–61) are sorted into the redundant group (maroon bars in Panel B) because each of them shares a common 5ʹ or 3ʹ end with a longer fragment. In a similar process as we described in previous section, a total of eleven multi-fragment islands are then formed and marked in dashed boxes in Fig. 4b. Then, by following the rules of exclusivity and greediness, Islands a, b, d, e, g, h, j, and k are assigned to Molecule 1 (colored in orange), and Islands c, f, and j are assigned to Molecule 2 (colored in blue). Next, Fragments 3, 11, 12, and 13 are assigned to Molecule 1 and Fragment 50 joins Molecule 2.

The BIGseq algorithm further identified that Fragment 34 has three extra bases, CTG, at its 3′ end. These bases should be trimmed because they are the last three bases of the primer. Removing these three bases allows Fragment 34 to chain with Island b through a canonical junction. In the final step, Molecule 1 is set between 183,025 and 192,112 and Molecule 2 is set between 185,347 and 190,594, as shown in Panel C, to conclude the reconstruction of two homologous mono-ploid molecules. At the completion, Region 2 has two copies, Region 1 and Region 3 have one copy, and areas 3′ immediately adjacent to region 1 and 5′ to Region 3 have 0 copies (Fig. 4c).

Several points need to be stressed: (a) it is unambiguous that Fragments 19–26 originated from one molecule, referred to as “in phase,” as they are chained by junctions (Fig. 4b). 2) The same is not necessarily true for Fragments 1–34 because there are gaps between Fragments 3 and 4, 18 and 19, 28 and 29, 30 and 31, 45 and 46, and 49 and 50. (b) Although there are gaps between Fragments 8 and 9, 10 and 11, 11 and 12, 12 and 13, 13 and 14, 38 and 39, it is of high confidence that Fragment 34 and Fragments 4–18 originate from one haploid molecule while Fragments 35–45 originate from a different molecule. Although neither haploid molecule is contiguously covered, BIGseq is the first technology to enable physical connections to be established through the rule of exclusivity.

## Discussion

Single-cell DNA sequencing methods are often treated as bulk sequencing methods that are merely applied to single cell samples. The apparent similarity of the sample preparation and processing procedures shared by single cell and bulk sequencing protocols may reinforce this notion. For instance, similar to a standard Nextera bulk sequencing protocol^15^, BIGseq also utilizes the following sample prep and processing procedures:

1. Tagmentation of genomic DNA to construct the primary library,
2. Amplification of the library,
3. Sequencing the library using NGS,
4. Mapping and deduplication and,
5. Haploid analyses.

However, due to the heterogeneity of gene copy numbers in cancer cells and the undefined number of genomes in the bulk sample, the copy number per genome determined by Nextera-prepared bulk genomes may be calculated to be a decimal, a number that does not exist in nature and creates a conundrum to make sense at the biological level. However, copy numbers can be determined discretely when each of the genomes is interrogated individually. Unfortunately, current single cell DNA sequencing technologies fail to capture the discreteness, as they adopt secondary analysis methodology developed for bulk sequencing^12^. These statistics-heavy methodologies are not robust; the inferred copy number varies when a different bin size is chosen, or a different segmentation option is selected, or ploidy of the genome deviates from diploid^12^. In contrast, we have taken a different bioinformatic approach which is built upon the deterministic array of fragments generated by tagmenting a stretch of DNA. It simply pieces those fragments back into a single molecule by mapping and concatenating through junctions. Along with its simplicity, this approach carries with it the ability to count DNA molecules and identify mutations at single base resolution to specific haploids.

Our new approach can be applied to the studies of normal single cells of human origin, whether they are haploid, diploid, tetraploid, etc., and cancer cells with altered ploidities as on microscopic level, the sequencing data set from single human genome is expected to be similarly structured as the yeast counterpart as we have shown in this manuscript. The complexity in human genome may introduce additional challenges and inspire new innovations. Subsequent calibration of the performance of the BIGSeq against human single cells of known ploidy will illustrate the power and resolution of this method.

## Supplementary Information

Supplementary Information.
