# Supplementary material for: Reconstructing and counting genomic fragments through tagmentation-based haploid phasing
Source: Sci Rep. 2021 Sep 23;11:18907. doi: 10.1038/s41598-021-97852-w (PMC8460729; doi:10.1038/s41598-021-97852-w)

Supplementary Fig. 1. Leftover primer sequence. Sequences 1–10 are the reverse reads for Fragment 19 with coordinates 658,201-658,361 of Chr XIV/NC_001146.8, while Sequences 11–18 are the forward reads for Fragment 20 (658,350–658,629). The subsequence CAG from 11 to 18 at the very 5ʹ end are not present in the reference genome or Fragment 19. They are the last three bases from primers.


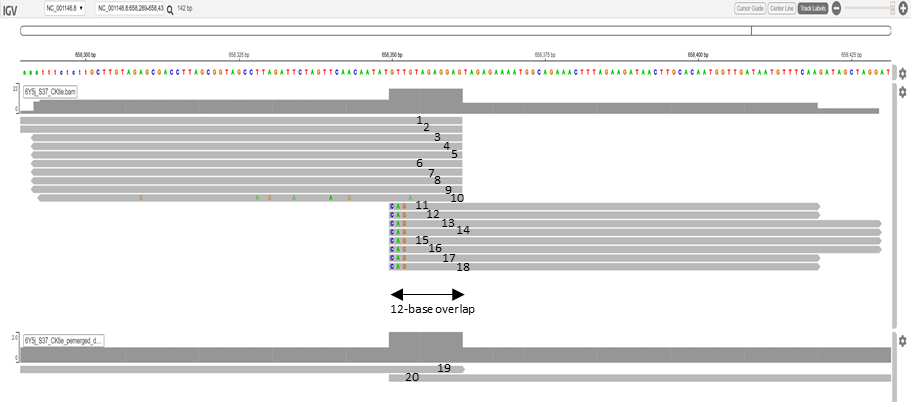


Supplementary Fig. 2. Examples of Complex Scenarios of 00/11 exhibited by Fragments 1–3 (A), 11/00 exhibited by Fragments 1–3 (B), 01/10 exhibited by Fragments 1–3 (C), and 10/01 exhibited by Fragments 1 and 2 (D) respectively.


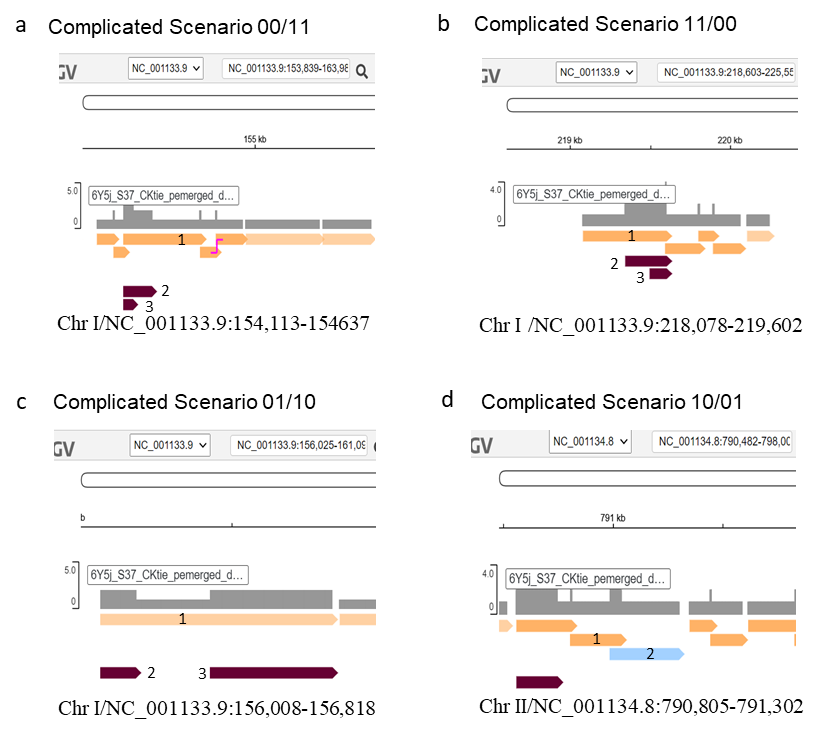

Supplement: Supplementary file 1 — Supplementary Information. [file 41598_2021_97852_MOESM1_ESM.docx]
